# Emergent variant modeling of the serological repertoire to norovirus in young children

**DOI:** 10.1016/j.xcrm.2023.100954

**Published:** 2023-02-27

**Authors:** Lisa C. Lindesmith, Paul D. Brewer-Jensen, Helen Conrad, Kathleen M. O’Reilly, Michael L. Mallory, Daniel Kelly, Rachel Williams, W. John Edmunds, David J. Allen, Judith Breuer, Ralph S. Baric

**Affiliations:** 1 Department of Epidemiology, University of North Carolina at Chapel Hill, Chapel Hill, NC 27599, USA; 2 Centre for Mathematical Modelling of Infectious Diseases and Department of Infectious Disease Epidemiology, London School of Hygiene and Tropical Medicine, London WC1EW 7HT, UK; 3 Department of Infection Biology, Faculty of Infectious and Tropical Diseases, London School of Hygiene and Tropical Medicine, London WC1E 7HT, UK; 4 Department of Infection, Immunity and Inflammation, UCL Great Ormond Street Institute of Child Health, University College London, London WC1N 1EH, UK; 5 Department of Genetics & Genomic Medicine, UCL Great Ormond Street Institute of Child Health, University College London, London WC1N 1EH, UK; 6 Department of Microbiology, Great Ormond Street Hospital for Children NHS Foundation Trust, London WC1N 3JH, UK

**Keywords:** norovirus, neutralizing antibodies, blockade antibodies, antigenic cartography, variants of concern, antigenic seniority, immune imprinting

## Abstract

Human norovirus is the leading cause of acute gastroenteritis. Young children and the elderly bear the greatest burden of disease, representing more than 200,000 deaths annually. Infection prevalence peaks at younger than 2 years and is driven by novel GII.4 variants that emerge and spread globally. Using a surrogate neutralization assay, we characterize the evolution of the serological neutralizing antibody (nAb) landscape in young children as they transition between sequential GII.4 pandemic variants. Following upsurge of the replacement variant, antigenic cartography illustrates remodeling of the nAb landscape to the new variant accompanied by improved nAb titer. However, nAb relative avidity remains focused on the preceding variant. These data support immune imprinting as a mechanism of immune evasion and GII.4 virus persistence across a population. Understanding the complexities of immunity to rapidly evolving and co-circulating viral variants, like those of norovirus, severe acute respiratory syndrome coronavirus 2 (SARS-CoV2), and dengue viruses, will fundamentally inform vaccine design for emerging pathogens.

## Highlights

• Sera from young children block binding of antigenically diverse GII.4 noroviruses
• GII.4 variant replacement shapes population serological antibody titer over time
• High-avidity antibody remains focused on previous, antigenically related GII.4 variants
• Immune imprinting molds GII.4 antibody responses and virus emergence potential

## Graphical abstract

**Figure:**
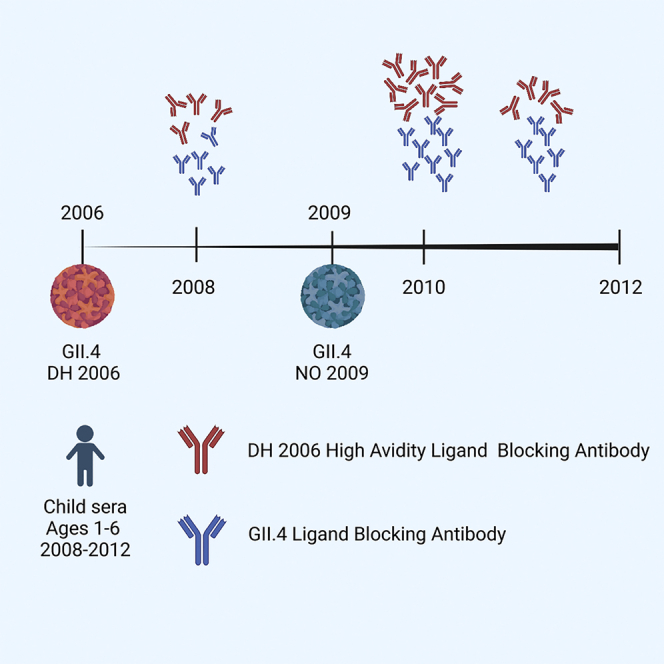

## Teaser

Norovirus GII.4 variants are a leading cause of gastroenteritis in children. Lindesmith et al. demonstrate that an emergent variant remodels antibody titer, while high-avidity antibody remains focused on the preceding related variant. These data support immune imprinting as a mechanism of GII.4 immune evasion and virus persistence.

## Introduction

Globally, diarrheal diseases account for one in nine deaths in young children, more than HIV, malaria, and measles combined.^1^^,^^2^ In countries where rotavirus vaccination has been implemented, human norovirus has become the leading cause of acute nonbacterial gastroenteritis,^3^^,^^4^^,^^5^^,^^6^^,^^7^ resulting in more than 200,000 deaths per year.^8^ Norovirus infection in children induces more frequent vomiting episodes over a longer duration of time than bacterial gastroenteritis,^9^^,^^10^^,^^11^ and children less than five years old are the most likely to suffer severe disease.^12^^,^^13^^,^^14^^,^^15^^,^^16^ In lower- and middle-income countries, ∼14% of pediatric gastroenteritis is caused by norovirus.^17^ In the United States, norovirus was the most frequently detected pathogen in medically attended acute gastroenteritis (MAAGE), and children less than five years old were the most likely to suffer from MAAGE.^12^ These global disease burden numbers are similar to those for rotavirus before vaccine implementation,^18^^,^^19^^,^^20^ emphasizing the need for development of a norovirus vaccine.

The primary challenge to development of a norovirus vaccine is viral diversity.^21^^,^^22^^,^^23^^,^^24^^,^^25^ Human noroviruses are currently divided into 10 genogroups (GI–GX) and further subdivided into more than 35 genotypes.^26^ Despite tremendous viral diversity, variants within the GII.4 genotype cause 50%–70% of all norovirus outbreaks worldwide.^27^^,^^28^^,^^29^ Recurrent GII.4 pandemics were recorded in 1995 (Grimsby variant), 2002 (Farmington Hills), 2004 (Hunter), 2006 (Den Haag), 2009 (New Orleans), and 2012 (Sydney). Until the Sydney (SY) 2012 pandemic, each pandemic variant circulated for two to five years before being replaced by its successor. SY 2012 continues to be the dominant global variant in 2022.^30^ The reasons for the shift from regular variant transitions to one dominant variant since 2012 are not well understood, although immune imprinting has been proposed to contribute to SY 2012 persistence.^31^^,^^32^

Immune imprinting describes the inherent predisposition to preferentially activate pre-existing memory B cells over naive B cells upon subsequent exposure to a similar virus. Preferentially activating a memory response has the benefit of quick production of high-affinity antibodies but can delay production of sterilizing antibodies when the current infecting strain is divergent from the original strain at protective antibody epitopes. This phenomenon was first described for influenza viruses.^33^^,^^34^^,^^35^ Current technologies capable of deconvoluting epitope molecular signatures within the serum antibody repertoire indicate that immune imprinting may be a common phenomenon in antibody responses to highly adaptable viruses, including HIV,^36^ dengue,^37^ severe acute respiratory syndrome coronavirus 2 (SARS-CoV-2),^38^ and human norovirus,^32^^,^^39^^,^^40^ all RNA viruses with a high propensity for immune evasion driven by changes in neutralizing antibody epitopes.

The effect of immune imprinting on antibody responses at the population level can be visualized via antigenic cartography, an analytical pipeline developed by Smith et al.^41^ to visualize the antigenic relatedness of a series of influenza A viruses. This method charts the position of a panel of serum and virus variants relative to each other based on antibody responses (neutralizing antibody [nAb], avidity), revealing patterns of population exposure and variant cross-reactivity. Recently, the methodology has been applied to additional RNA viruses, including dengue,^42^ SARS-CoV-2,^43^ and human norovirus,^32^^,^^44^^,^^45^ allowing visualization of the antigenic relationship across multiple variants at the population level and how these relationships change over time and exposure, key metrics for informing vaccine development.

Norovirus pandemic variant emergence correlates with evolution in key antigenic sites on the surface of the viral capsid protein, with limited cross-neutralization between successive variants.^23^^,^^24^^,^^46^ However, capsid amino acid sequence similarity and antigenic cartography illustrate a difference in the degree of antigenic relatedness between variants that circulated before and after Den Haag (DH) 2006. Whereas the variants that circulated post DH 2006 are more closely related to DH 2006 and to each other, the variants that circulated before DH 2006 are less closely related to any other variant.^25^^,^^45^^,^^47^ The mechanisms that allowed epidemiological success of the New Orleans (NO) 2009 and SY 2012 variants following the antigenically related DH 2006 variant are unclear.

In adults, antibody responses to GII.4 variants are confounded by pre-existing cross-reactive antibody responses to previous variants via antigenic seniority, a form of immune imprinting characterized by hierarchical back-boosting of antibodies to ancestral variants upon exposures to related variants.^39^^,^^40^ Immune imprinting patterns are associated with infection outcomes to influenza A viruses^33^ and will likely drive responses to SARS CoV-2 infection and vaccination^48^^,^^49^^,^^50^ as well as norovirus.^39^^,^^40^ The primary target populations for a norovirus vaccine are young children and the elderly because globally, these groups suffer the most severe disease and likelihood of death.^51^ Norovirus immune imprinting is likely established in childhood.^31^^,^^33^ How differences in variant exposure between children and the elderly over time impacts immune imprinting and response effectiveness to human norovirus is unknown but key for implementing effective vaccines for both groups.

To begin to understand the development of antibody-mediated immunity to human norovirus GII.4 variants, we characterized nAb responses in ∼700 young children, focusing on the period during the transition from DH 2006 to NO 2009 prevalence. Utilizing a surrogate neutralization assay that correlates with an *in vitro* virus neutralization assay and is a proposed correlate of protection,^39^^,^^52^^,^^53^^,^^54^ we determined the seroprevalence of the GII.4 nAb in children by age, year of serum collection, and dominant circulating GII.4 variant. In this large-scale longitudinal study of nAb responses to norovirus in children, we identify serological evidence of genetic resistance to GII.4 infection, chart the development of population-level immunity to an emergent pandemic variant, map the corresponding remodeling of the serological nAb repertoire in young children, and identify a signature of immune printing in nAb responses.

## Results

### Seroprevalence to GII.4 norovirus variants in young children

**Figure 1:**
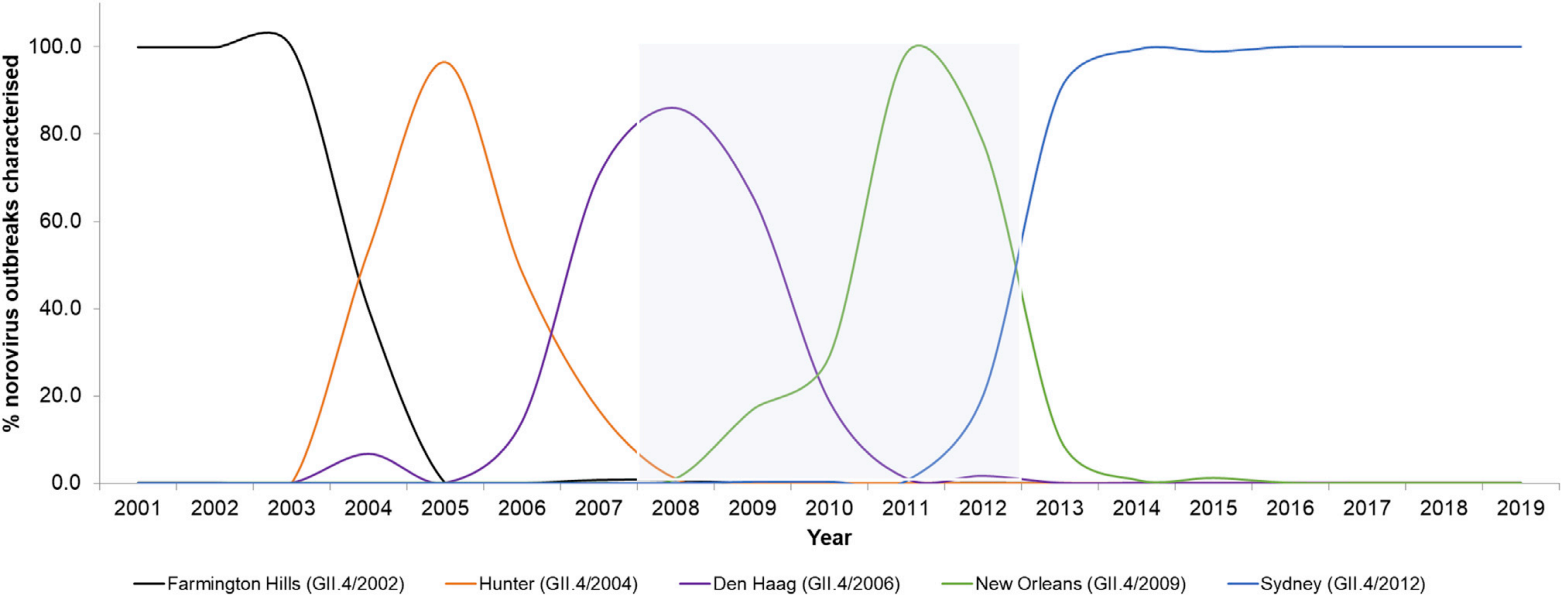
GII.4 human norovirus pandemic variants Shown are serial pandemic GII.4 variants (colored curves) as a percentage of documented norovirus outbreaks over time in England (GOV.UK official statistics: Summary of Norovirus surveillance 2018 to 2019). In this study, sera were collected between 2008 and 2012 (blue shading) from 686 children aged one to six years and tested against a panel of virus-like particles (VLP) representing the time-ordered GII.4 pandemic variants FH 2002, DH 2006, NO 2009, and SY 2012. See also Figure S1.

For each year between 2008 and 2012, sera from 686 children ages one to six years and living in the United Kingdom were stratified into groups of approximately 25 children of each age (686 total children) and were analyzed for nAbs to a panel of time-ordered GII.4 pandemic variants in a surrogate neutralization assay (Table 1). The distribution of available samples was similar between years and ages, with the exception of samples from ages one and six years being fewer for 2012. The GII.4 variants selected for the study included pandemic variants that were in circulation during sample collection: DH 2006 (circulation 2006–2011), NO 2009 (circulation 2009–2012), and SY 2012 (circulation 2012–2022) (Figure 1). An additional pandemic variant, Farmington Hills (FH 2002) (circulation 2002–2005), was included for comparison of a more antigenically diverse GII.4 variant (Figure S1). FH 2002 predominated before most of the children were born; thus, children were less likely to have been exposed to this variant. Serum collection between 2008 and 2012 represent population immunity during the transition from DH 2006 to NO 2009 as the dominant variant.

**Table 1:** Seroprevalence study sample set by age of child and year of sample collection

Age	2008	2009	2010	2011	2012	Total
1.0–1.9	23	24	25	24	7	103
2.0–2.9	25	24	23	23	12	107
3.0–3.9	25	25	24	24	23	121
4.0–4.9	25	25	25	25	24	124
5.0–5.9	25	25	25	23	24	122
6.0–6.9	24	24	25	25	11	109
Total	147	147	147	144	101	686

**Figure 2:**
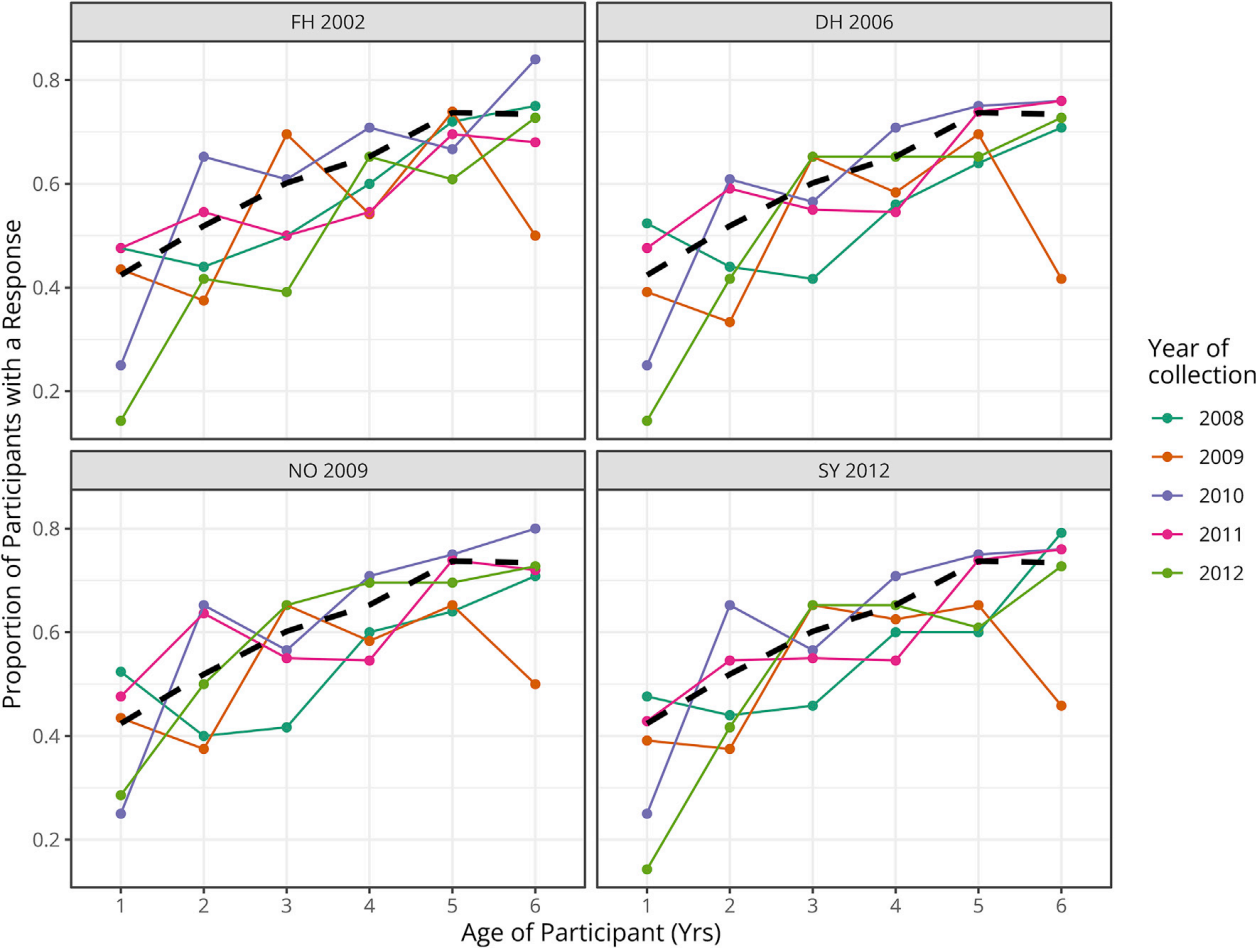
Serological evidence of GII.4 exposure in young children The proportion of participants with titer (ID50 ≥ 10) to a specific GII.4 variant was determined by age, year, and variant in sera collected from children aged one to six years between 2008 and 2012. Only sera tested against all four variants were included in the analyses (n = 656). The dashed line represents the proportion of responders in all years of sample collection combined. See also Figure S2 and Table S1.

For all years combined, including only sera with sufficient volume for testing of all four GII.4 variants (n = 656, 96%), the average percentage of children with a detectable nAb titer (inhibitory dose 50% titer [ID50] ≥ 10) to any tested GII.4 variant generally increased with age, regardless of collection year or GII.4 variant tested. Fitting a catalytic model^55^ to these data suggests that children develop an nAb response to norovirus at a rate of 1.08 per year (95% confidence interval [CI], 0.72–1.51) and, on average, 30% (24%–36%) of children are likely to never respond to any GII.4 variant (Figures 2 and S2; Table S1). These data are consistent with previous findings using other approaches that demonstrate that GII.4 susceptibility is mediated by host genetics, specifically a functional *FUT 2* gene that defines a “secretor” phenotype.^56^ Although the secretor status of these children is unknown, ∼20% of northern European populations do not have a functional *FUT 2* gene and would likely be resistant to GII.4 infection.^57^ In addition to genetic susceptibility, antibody titers waning to below the limit of detection or targeting other GII.4 variants not detected by surveillance as well as lack of exposure to norovirus in some children are likely to account for a percentage of the non-responders. To minimize the confounding influence of genetic resistance on the study outcomes, only sera with an nAb titer above the limit of detection to at least one GII.4 variant were included in further analyses.

**Figure 3:**
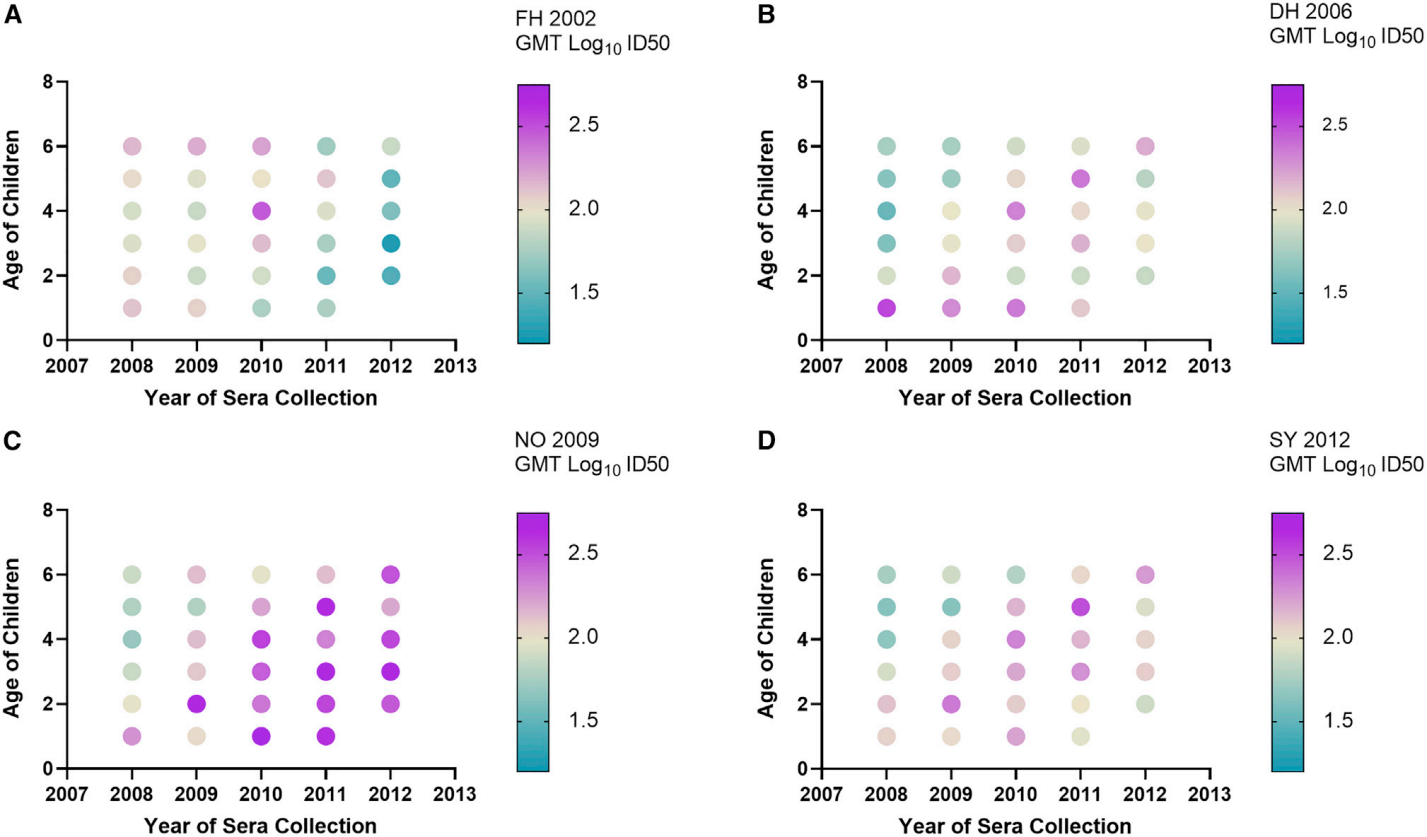
GII.4 nAb by year of sample collection, age of child, and GII.4 variant (A–D) Each marker represents the GMT of the log_10_-transformed ID50 for children of that age (see also Table S2) for pandemic GII.4 variants FH 2002 (A), DH 2006 (B), NO 2009 (C), and SY 2012 (D). Sera were tested once per VLP with internal and plate controls. Tests were repeated when a control failed. Only sera from children who had an nAb titer (ID50 ≥ 10) to at least one GII.4 variant were included in the analyses (n = 423, 62% total). Log_10_ GMT are color coded from lowest (teal) to highest (purple).

Among children with GII.4 nAb (n = 423, 62%, Table S2), response trends were evident across year of sample collection, age of child, and variant tested (Figure 3). To compare nAb breadth by age and circulating variant, we first compared the antibody responses to each variant stratified by age and year of sample collection. Although the small number of age-stratified responders limited our ability to detect a significant response to any one variant by year of age, distinct antibody patterns were apparent. At each year of collection, sera from one-year-old children were able to neutralize antigenically diverse variants, including variants not known to be circulating at the time of serum collection (Figure 3). This cross-nAb activity extended to FH 2002, a variant thought to be largely extinct before most of the children sampled in this study were born (Figure 1), supporting potential development of cross-nAb early in life and after a limited number of exposures, although specific variant exposure cannot be ruled out in a seroprevalence study. Antibody titers in older children were also cross-neutralizing between distinct pandemic variants with little correlation between age of child and variant response (Figure 3). The geometric mean titer (GMT) to NO 2009 in older age groups climbed in 2010–2011 and remained high in 2012. Antibody titer to DH 2006 and SY 2012 showed similar trends as NO 2009, being highest initially in younger age groups and rising over time in older ages. In contrast, antibody titer to FH 2002 trended high in all age groups in 2008 and thereafter declined, first in younger and subsequently in older age groups (Figure 3). Together, these data indicate that GII.4 immunity is cross reactive among variants.

### Immune imprinting shapes GII.4 high-avidity nAb responses in young children

**Figure 4:**
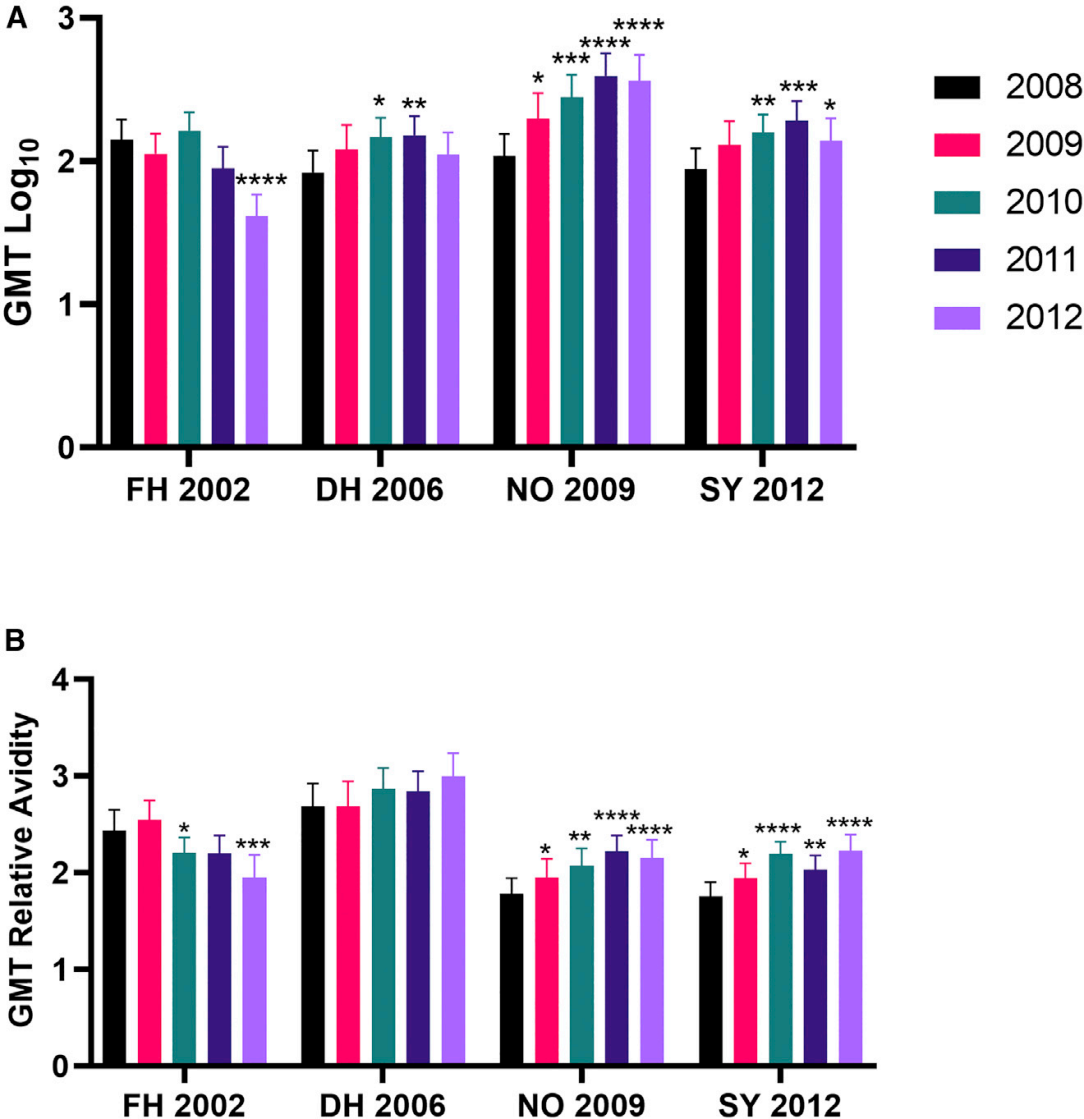
NO 2009 nAb is most abundant, while the high-avidity DH 2006 nAb persists post NO 2009 emergence (A) The GMT ID50 of GII.4 nAbs in sera from children with ID50 above the limit of detection (≥10) to any GII.4 variant (n = 423) was compiled by year of sample collection and GII.4 variant. (B) GMT relative Ab avidity, as measured by the slope of the Ab neutralization curve of positive titer samples. Sera were tested once per VLP with internal and plate controls. Tests were repeated when a control failed. Error bars, 95% CI. ∗, significantly different from 2008 for each virus, Mann-Whitney test. ∗p ≤ 0.05, ∗∗p ≤ 0.01, ∗∗∗p ≤ 0.001, ∗∗∗∗p ≤ 0.0001. See also Table S2.

Next, we compared the antibody responses by year of sample collection with all age groups combined to evaluate overall trends in antibody titer and cross-reactivity (Figure 4). Child population nAb titers were similar between NO 2009 and the other variants in years 2008 and 2009 (ANOVA, Kruskal-Wallis multiple comparisons test), indicating that a basal level of cross-GII.4 immunity persists during non-variant surge years, as described for adults^58^^,^^59^ (Figure 4A). Corresponding with the replacement of DH 2006 with NO 2009 as the global dominant GII.4 variant between 2009 and 2012, antibody titer to NO 2009 rose markedly, with GMT increasing 1.8-fold in 2009 (predominantly led by children two years old or younger (Figure 3), 2.6-fold in 2010, and holding at ∼3.5-fold above the 2008 titer in 2011 and 2012. As expected for closely related variants, at the time of peak NO 2009 nAb titer in 2010–2012, the titer to DH 2006 rose 1.8-fold in 2010 and 2011 before returning to 2008 levels in 2012. Concurrently, the SY 2012 titer increased 1.8-fold in 2010, 2.2-fold in 2011, and 1.6-fold in 2012. The nAb GMT titer was similar each year between DH 2006 and SY 2012 (Mann-Whitney test comparing each year), indicating that NO 2009 nAbs are similarly cross-reactive to DH 2006 and SY 2012 and/or that some antibodies (Abs) neutralize DH 2006 and SY 2012 variants similarly. nAb GMT to NO 2009 was higher than DH 2006 and SY 2012 in 2010–2012 (ANOVA, Kruskal-Wallis multiple comparisons test). In contrast, nAb GMT against the more divergent variant FH 2002 did not increase with NO 2009 emergence (Figure 4A). Titers to FH 2002 remained consistent through 2011 before decreasing by 3.5-fold in 2012 compared with 2008.

**Figure 5:**
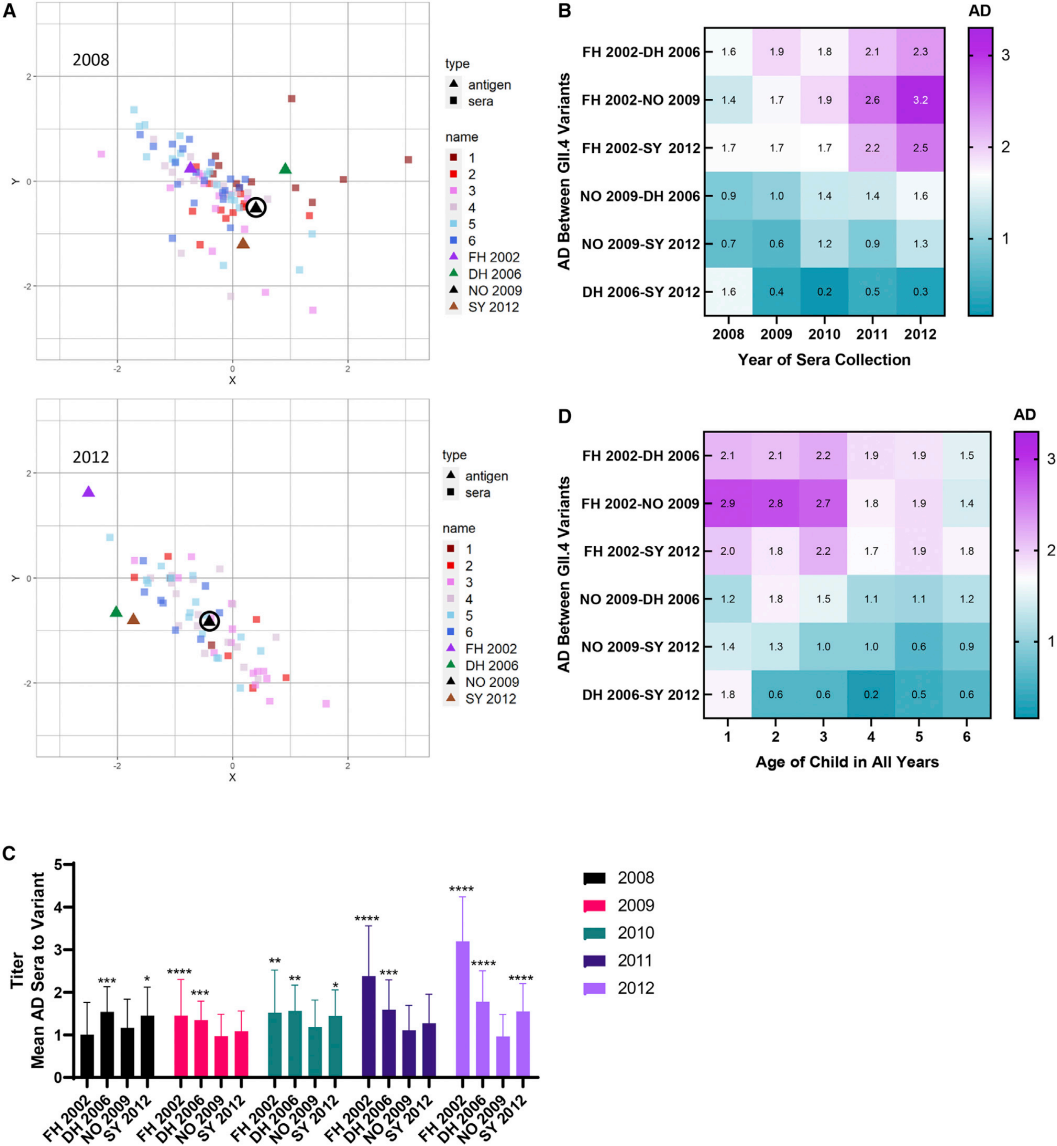
The serological nAb repertoire titer is remodeled over time in response to a new dominant variant (A) The antigenic relationship between GII.4 variants (triangles) relative to the nAb response in each child’s serum sample with a titer to at least two GII.4 variants reported (squares, n = 413; Table S3), color coded by child year of age (color of squares: 1, dark red; 2, red; 3, pink; 4, light pink; 5, light blue; 6, dark blue), was mapped via antigenic cartography for 2008 and 2012. One grid box (antigenic distance [AD] unit) corresponds to a two-fold change in titer. As a visual marker, NO 2009 is circled. (B) Summary AD between variants over time, color coded from most (teal) to least (purple) closely related. (C) Mean AD between sera and variants by year. Error bars, SD. ∗p ≤ 0.05, ∗∗ p ≤ 0.01, ∗∗∗p ≤ 0.001, ∗∗∗∗p ≤ 0.0001 compared with NO 2009 for each year of serum collection by one-way ANOVA Dunnett multiple comparisons test. (D) Summary AD between variants by age of child at serum collection for all years combined, color coded from most (teal) to least (purple) closely related. See also Figure S3 and Table S3.

To further explore the influence of GII.4 variant exposure on the nAb landscape, antigenic cartography was employed to visualize the change in the antigenic relationship between GII.4 variants before, during, and after the transition from DH 2006 to NO 2009 as the dominant GII.4 variant (Figures 5A and S3). The serum nAb titer in children between 2008 and 2012 increasingly differentiated FH 2002 from the other variants (Figures 5B and S3), as the antigenic distance (AD) between FH2002 and other variants increased over time. The AD between FH 2002 and NO 2009 increased from 1.4 in 2008 to 3.2 in 2012, the highest AD identified among the variants across the period. In contrast to the increasing distances between NO 2009 and FH 2002 over time, the distance between NO 2009 and DH 2006 or SY 2012 increased less than one unit (corresponding to a <two-fold change in titer) between 2008 and 2012 (Figures 5B and S3), confirming the high degree of antigenic similarity between these three variants. Notably, the AD between DH 2006 and SY 2012 decreased with the emergence of NO 2009, diminishing from AD 1.6 in 2008 to 0.39 in 2009 and remaining low through 2012 (range, 0.17–0.47). These data indicate that the similar titers between DH 2006 and SY 2012 reported in Figure 4A are the result of shared nAb epitopes between DH 2006 and SY 2012; i.e., that these variants are antigenically more similar to each other as opposed to equally different from NO 2009.

Next, we compared the mean AD between the sera and each GII.4 variant over time. As population immunity develops to an emergent variant, the lowest mean AD would be expected to shift to the new penetrant variant (NO 2009 post 2010). Accordingly, in 2011 and 2012, the AD was lowest between the serum mean and NO 2009 (Figure 5C). In 2012, the AD between the serum mean was 0.96 for NO 2009, 1.8 for DH 2006, and 1.5 for SY 2012, indicating that child population nAb responses remained highly cross reactive between DH 2006, NO 2009, and SY 2012 through 2012 (Figure 5C). Conversely, the mean AD between the sera and FH 2002 increased from 1.0 in 2008 to 3.2 in 2012, as population immunity to FH 2002 declined. Notably, at each year, the mean AD between the sera and DH 2006 and NO 2009 was significantly different, supporting the antigenic differences between these sequential GII.4 variants.

The AD between the GII.4 variants differed between age groups across years (Figure 5D). Only sera from one-year-old-children clearly differentiated DH 2006 from SY 2012. Sera from children aged three years or younger differentiated between variants, particularly FH 2002, more clearly than sera from children four to six years old. Sera from children aged four years or older were highly cross reactive to DH 2006, NO 2009, and SY 2012 (all AD ≤ 1.2). These data indicate that several years of virus circulation post new variant emergence occurred before the child population serological nAb repertoire was significantly focused on NO 2009 and that sera nAb responses likely broaden with child age and GII.4 variant exposure.^31^^,^^32^

Before and after NO 2009 nAb repertoire reprogramming, population immunity continued to be highly cross-reactive with DH 2006, NO 2009, and SY 2012. However, this level of cross-reactivity was likely not protective against subsequent variant infection with NO 2009 or SY 2012 as each virus emerged in this demographic group, suggesting that a metric other than nAb titer may be needed to predict protection from infection from closely related antigenic variants. Serological studies for SARS-CoV-2 and HIV, additional viruses with many antigenic variants, have found the slope of the neutralization curve to predict Ab breadth and epitope specificity and to discriminate between closely related variants.^60^^,^^61^^,^^62^^,^^63^^,^^64^ In a previous study, we demonstrated that Ab avidity correlated with the slope of the neutralization curve and that norovirus infection increased nAb titer to multiple genotypes but only increased the slope of the neutralization curve to the infecting genotype,^65^ supporting observations that slope could be a surrogate for nAb specificity between antigens with common neutralizing epitopes. Theoretically, the slope of the neutralization curve should approximate 1. Slopes greater than 1 indicate cooperative binding and/or favorable Ab-epitope kinetics that increase neutralization.^63^ To further explore the quality, not only the quantity, of the nAb response to the GII.4 variants, we analyzed the nAb curve slope^65^ to all variants with a titer above the assay limit of detection (Figure 4B, Table S2). Because we did not differentiate between nAb cooperativity and kinetics in this study, we will refer to the complex serum components’ effect on the slope of the neutralization curve as a measure of nAb relative avidity.^65^ nAb relative avidity to FH 2002 and DH 2006 was high (GMT Hill slope 2.4–2.7, respectively) pre-NO 2009 circulation, indicating the persistence of the high-relative-avidity GII.4 nAb to ancestral GII.4 variants in children, as described previously for adults.^65^ Following NO 2009 replacement of DH 2006 (2010–2012), relative avidity to NO 2009 and SY 2012 improved modestly, ranging from 2.0–2.2 across years. While this is a statistically significant increase in relative avidity compared with 2008, nAbs to NO 2009 or SY 2012 did not reach the quality of pre-existing nAbs to DH 2006. DH 2006 relative avidity remained the highest over time and did not change in response to emergence of NO 2009 (range, 2.7–3.0 each year) (Figure 4B). In contrast, nAb relative avidity to FH 2002, the predominant global variant between 2002 and 2004, began to trend downward in 2010 and continued to decline, reaching 2.0 in 2012 (Mann-Whitney test). These data suggest that, after two to three years of DH 2006 penetrance in the population, immunity to DH 2006 is likely driven by high-affinity nAbs as opposed to high titers of less potent nAbs, as described for NO 2009.

**Figure 6:**
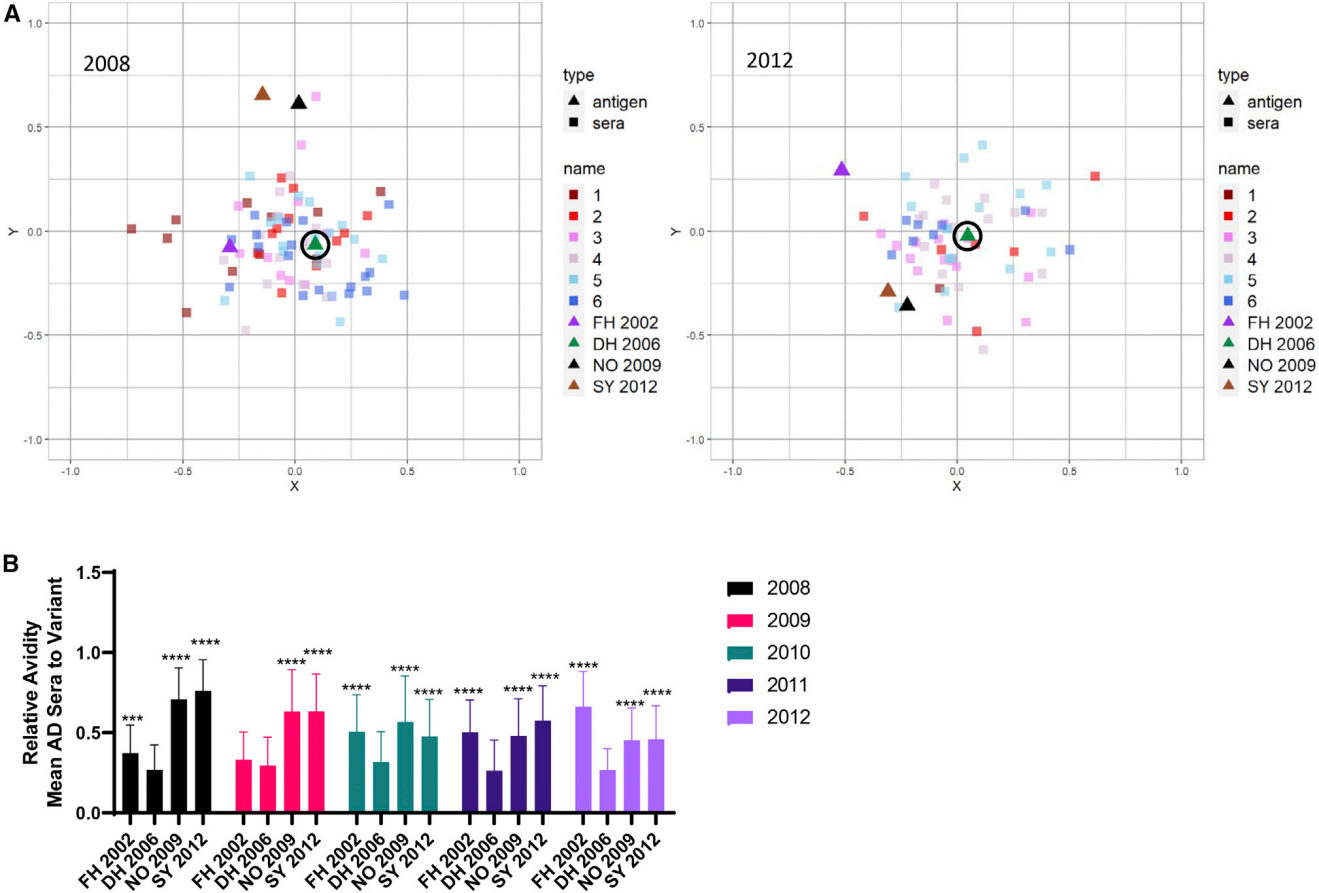
nAb relative avidity is shaped by immune imprinting from previous norovirus variants (A) The antigenic relationship between GII.4 variants (triangles) relative to the nAb relative avidity in serum from each child with a measurable titer to at least two GII.4 variants (n = 394; Table S4) (squares), color coded by year of age (color of squares: 1, dark red; 2, red; 3, pink; 4, light pink; 5, light blue; 6, dark blue) was mapped via antigenic cartography for 2008 and 2012. One grid box (AD unit) corresponds to a two-fold change in avidity. As a visual marker, DH 2006 is circled. (B) Relative avidity mean AD between variants and the serum mean in (A) and (B) and Figure S4. Error bars, SD. ∗, different from DH 2006 by one-way ANOVA Dunnett multiple comparisons test. ∗∗∗p ≤ 0.001, ∗∗∗∗p ≤ 0.0001. See also Figure S4 and Table S4.

Antigenic cartography comparing the AD between the mean of the sera and each variant based on the relative avidity of the nAb support immune focusing of high-avidity nAbs on DH 2006 across the study period. In 2008, the mean AD between the sera and the GII.4 variants based on nAb relative avidity was least for DH 2006 (AD 0.3) and FH AD 2002 (0.4) (Figures 6A and 6B). For nAb relative avidity, the mean AD between the sera and DH 2006 remained 0.3 in years 2009–2012, regardless of the higher nAb titers to NO 2009 (Figures 6A, 6B, and S4). After 2009, the mean AD between sera and variant increased to FH 2002 and decreased for NO 2009 and SY 2012 (Figure S4) (one-way ANOVA). These data indicate that, during NO 2009 emergence and dominance, unlike nAb titer, nAb relative avidity remained focused on DH 2006 in these children, a signature of immune imprinting of previous norovirus infection on nAb responses to current norovirus infection.

Together, nAb titer and relative avidity analyses describe a multiphasic remodeling of the serological repertoire in response to norovirus infection. The repertoire is comprised of waning responses to some variants, mounting responses to an emergent variant, and compounded by significant cross-reactivity between closely related variants. Although the nAb directed to NO 2009 is most abundant in sera, the NO 2009 nAb may not be the most efficient at neutralizing virus. Continued maintenance of the high-relative-avidity nAb to DH 2006 throughout the NO 2009 circulation period would likely suppress re-emergence of DH 2006. Conversely, given the extensive cross-reactivity between DH 2006 and NO 2009/SY 2012, the high-relative-avidity nAb to DH 2006 may have negatively impacted development of high quality nAb to either of those variants. High-avidity DH 2006 Abs may bind to NO 2009 and SY 2012 without providing potent neutralization, as shown for some mAbs,^25^ potentially contributing to immune escape of these similar variants.

## Discussion

In 2008, we proposed a mechanism of GII.4 variant persistence in human populations based primarily on virus evolution at key nAb epitopes leading to immune escape.^23^ Here, in this comprehensive GII.4 nAb immunity study undertaken, we identified immune imprinting in nAb responses to GII.4 variants in children, specifically within the contemporary GII.4 variants DH 2006, NO 2009, and SY 2012. These data, coupled with recent findings demonstrating immune imprinting as a determinant of GII.4 nAb responses in adults,^32^ indicate that the mechanism of GII.4 immune evasion has evolved over time, likely in response to acquired population immunity. Clear antigenic differentiation occurred between the early GII.4 pandemic variants US95/96, FH 2002, and DH 2006, and these differences indicate immune escape as the mechanism of viral emergence and dominance.^23^^,^^46^^,^^58^^,^^66^ However, immune escape is unlikely to fully explain NO 2009 or SY 2012 emergence. Instead, our data suggest that immune imprinting drives a second mechanism of GII.4 evolution in human populations and likely contributed to the rapid succession of the antigenically similar variants DH 2006, NO 2009, and SY 2012.

Controlling infection in children is key to controlling norovirus spread. Therefore, an effective vaccine must provide durable protective immunity in a relatively naive population, likely by inducing broadly nAbs. Reported norovirus vaccine studies have focused on adults. However, prevalent norovirus-infecting genotypes, symptoms, and nAb responses may differ between adults and children,^31^^,^^67^^,^^68^^,^^69^ and children may immunologically respond differently to vaccination compared with adults.^70^^,^^71^ Thus, studies of responses in children should be prioritized to inform pediatric vaccine design.

A surprising finding here is the extensive GII.4 variant breadth of nAbs in the children. At the beginning of sample collection in 2008, DH 2006 had been the dominant GII.4 variant causing outbreaks for more than a year, NO 2009 and SY 2012 had not yet been widely detected, and FH 2002 was thought to have been extinct for several years.^24^^,^^72^ These observations are based on outbreak investigation data, which primarily include adults experiencing symptomatic infection,^72^ and are defined by qRT-PCR detection of virus in stool. Seroprevalence surveillance of nAbs to the GII.4 variants in young children generally supports these conclusions. Encompassing data on all infections (seropositivity), including the high percentage that are asymptomatic infections,^73^^,^^74^ may provide more information about the breadth of GII.4 viruses to which these children were exposed.

The identified cross-reactivity between NO 2009, DH 2006, and SY 2012 was expected based on previous studies of single-variant-immunized mice and infected humans.^25^^,^^44^^,^^45^^,^^47^ However, the observed high and persistent titer to FH 2002 in children’s sera collected between 2008 and 2010 was unexpected. FH 2002 is antigenically distant from the variants to which most children were thought to have been exposed during the study period.^44^^,^^66^ These titers may reflect some measure of continued circulation in young children of FH 2002 or antigenically similar viruses, even after growing population immunity decreased their prevalence in adults. Alternatively, FH 2002 may be particularly sensitive to cross-reactive nAbs. The eventual decrease in titer and relative avidity to FH 2002 by 2012 supports the former hypothesis. Importantly, these data provide evidence that very young children make Abs that may neutralize multiple GII.4 variants. When boosted through vaccination, these Abs could provide durable GII.4 protection.

Understanding the successive emergence of NO 2009 and SY 2012 during high population immunity to DH 2006 when these three variants are antigenically closely related is key to informing vaccine design to norovirus and other RNA viruses that undergo frequent variant replacements. Our findings here support similar conclusions found in studies of adults implicating immune printing as a driving factor in nAb responses to norovirus and influenza infection.^31^^,^^32^^,^^33^^,^^39^^,^^40^^,^^75^ While children mounted robust nAb titer increases to NO 2009 exposure, these nAbs were not as mature/effective at neutralization as pre-existing nAbs to DH 2006, as defined by differences in nAb relative avidity. High Ab avidity/affinity correlates with protection from infection with viruses, including RSV^76^ and SARS-CoV2.^77^^,^^78^ Here, we used the slope of the neutralization curve as a surrogate measure of relative avidity in polyclonal sera. Like affinity, the slope of the neutralization curve, here referred to as “relative avidity,”^65^ has been shown to associate with nAb epitope, breadth, potency, and differentiation of closely related viruses.^60^^,^^62^^,^^65^ In polyclonal serum, the relative avidity likely primarily represents the portion of total Abs targeting neutralizing epitopes.^60^^,^^61^

Between 2008 and 2012, the relative avidity of population nAb immunity was highest for DH 2006. Similarly, GII.4 vaccination in adults in 2010 preferentially recalled nAbs to the Grimsby pandemic variant of the mid-1990s.^40^ nAb titer was sufficient to differentiate the ancestral Grimsby variant from contemporary (2006–2009) variants, but nAb relative avidity was needed to discriminate among the contemporary variants in children. These data indicate that, in children between 2008 and 2012, NO 2009 infection recalled or maintained highly potent DH 2006 nAbs preferentially over NO 2009-specifc nAb, a signature of immune imprinting.

We further demonstrate that SY 2012 is antigenically more closely related to DH 2006 than NO 2009 in these young children. These serological findings do not agree with phylogenetic analysis of capsid protein sequence or with antigenic cartography using sera from mice immunized with single variants.^44^^,^^45^ Instead, these results suggest that DH 2006-initiated immune imprinting shapes the nAb response to NO 2009 as well as SY 2012 in children and adults. These findings could be confirmed by serological repertoire analyses.^39^^,^^79^^,^^80^^,^^81^ One would expect the most abundant monoclonal Abs in sera post SY 2012 exposure to preferentially target the ancestral US95/96 variant in adults, to target DH 2006 in children born 2006–2010, and to target SY 2012 in children born after 2012. Only one norovirus Ab repertoire analysis of adult sera from 2010 has been done, and this study supports US95/96 immune focusing.^39^ Immune imprinting can provide breadth of protection against antigenically similar viruses (such as influenza viruses of the same hemagglutinin subtype^33^ or between some SARS-CoV-2 variants^82^), or immune imprinting can be detrimental when poorly neutralizing, cross-reactive Abs are recalled in secondary exposures, as evidenced for dengue virus^83^ and here for norovirus. Additional studies of the adult repertoire with contemporary samples and similar studies of the child repertoire are needed to characterize how differences in exposure history may impact population immunity, either natural or vaccine induced.

nAb studies in adults and children tracking DH 2006 and SY 2012 responses support the above hypotheses^32^ but also point out key challenges, which may have applications to other rapidly evolving RNA viruses, like SARS-CoV-2.^48^^,^^49^^,^^50^ Even though these studies are limited by the lack of documented infection history or known secretor status of the children and use of cross-sectional rather than prospectively collected samples, the findings establish the conceptual framework for antigenic seniority in nAb responses to norovirus in young children. Consequently, the approach to vaccination, including long-term considerations regarding antigen selection, may need to be updated. Current vaccines in clinical trials include a single GII.4 capsid immunogen, either a consensus GII.4 based on extinct variants or SY 2012^84^ (NCT04854746). If the vaccine is given early enough to prevent most first symptomatic infections (∼six months of age),^85^^,^^86^ then the vaccine may guide the nAb response in children, as natural infection does in the absence of vaccination. Unfortunately, natural infection with a single variant does not provide durable protection from infection with emergent variants. Second-generation vaccines comprised of either multiple antigenically diverse GII.4 variants^87^ and/or immunogens with enhanced presentation of the highly conserved, less accessible GII.4 nAb epitopes^39^^,^^88^ may effectively boost broadly nAb in the short term and stimulate development of a rich pool of memory B cells recognizing multiple broadly neutralizing epitopes to provide long-term protection against emerging GII.4 norovirus variants. The low infectious dose of norovirus makes protection from infection difficult;^89^ however, there is evidence of Ab-mediated protection from severe symptoms.^90^^,^^91^ Relief of symptoms through vaccination would benefit the most vulnerable segments of societies, the elderly, and very young children.

### Limitations of the study

The primary limitations of this study are the lack of known norovirus genetic susceptibility and exposure history in the cohort. To mitigate the impact of these limitations, we down-selected samples to include only sera with a detectable titer to at least one GII.4 variant (genetically susceptible to GII.4 infection), and we presented key findings as aggregate responses over time with minimal subdivision into specific groups by age (minimizing differences in exposure across the population). Further, exposure to GII.4 variants in young children is unlikely to exactly overlap with the variant dominance reported in outbreak investigations (Figure 1), which primarily capture adult exposures. GII.4 variants may circulate earlier or for longer periods in young children.^92^ Older children within a serum year cohort may have had exposures to earlier variants and have detectable nAbs from those exposures. For example, the oldest children in 2008–2010 may have had infections with FH 2002 that are recorded here as cross-nAbs. Although serum from children with GII.4 exposure is highly cross-reactive among different strains of SY 2012,^31^ indicating that one sequence may adequately reflect the variant response, the sequences of the viruses to which children were exposed at any time is unknown and may diverge from the tested VLP at nAb epitopes. Finally, the constraint of limited sample volume of the archived children’s sera restricted our analyses to variant-specific nAb responses. The contribution of cross-genotype nAb, other GII.4 variant nAb, and non-neutralizing Ab responses should be considered in future studies to comprehensively characterize serological immunity. These studies would require either prospective studies that track exposure and responses or extensive panels of monoclonal Abs, goals outside the scope of this study.

## STAR★Methods

### Key resources table

**Table:** 

REAGENT or RESOURCE	SOURCE	IDENTIFIER
**Antibodies**		

Donkey anti-rabbit IgG-HRP	Cytiva	Cat# NA934

Bacterial and virus strains		
E.coli One Shot TOP10	Invitrogen	Cat# C404010

**Biological samples**		

Rabbit anti-norovirus capsid protein polyclonal serum	CoCalico Biologicals Custom Synthesis	N/A
Human sera	Public Health England Seroepidemiology Unit	N/A
Human stool	Public Health England Enteric virus unit	N/A

**Chemicals, peptides, and recombinant proteins**		

Cellfectin	Invitrogen	Cat# 10362100
FH 2002 Virus like particles	R. Baric, UNC-CH	GenBank: JQ478408
DH 2006 Virus like particles	R. Baric, UNC-CH	GenBank: JQ478409
NO 2009 Virus like particles	This study, D. Allen, LSHTM	GenBank: MZ_376650
SY 2012 Virus like particles	This study, D. Allen, LSHTM	GenBank: MZ_376651
Pig gastric mucin	Sigma-Aldrich	Cat# M1778
IGEPAL-CA630	Sigma-Aldrich	I8896-50ML
cOmplete™, EDTA-free Protease Inhibitor Cocktail	Sigma-Aldrich	Cat# 5056489001

**Critical commercial assays**		

mMESSAGE MACHINE T7 Transcription kit	Invitrogen	Cat#AM1344
Roche Expand High Fidelity System	Sigma Aldrich	11,732,641,001
SuperScript™ III Reverse Transcriptase System	Fisher Scientific	18,080,093

**Deposited data**		

ID50 data and calculated antigenic distances.	This paper.	https://zenodo.org/badge/latestdoi/518927355
Relative avidity (slope) data and calculated antigenic distances.	This paper.	https://zenodo.org/badge/latestdoi/518927355
NO 2009 Virus like particles	This study, D. Allen, LSHTM	GenBank: MZ_376650
SY 2012 Virus like particles	This study, D. Allen, LSHTM	GenBank: MZ_376651

**Experimental models: Cell lines**		

BHK cell line	ATCC	Cat# CCL-10
Sf9 cell line	Invitrogen	Cat# 11496015

**Oligonucleotides**		

ORF1/2-F1 CTG AG CAC GTG GGA GGG CG	ThermoFisher	N/A
TVN-Linker CGA CCT AGG TGA TAC ATG AT	ThermoFisher	N/A
TVN NTT TTT TTT TTT TTT TTT TTT CGA CCT AGG TGA TAC ATG AT	ThermoFisher	N/A
NO 2009 modification 1 AGATATCGAGCTCTATAAATAT GAAGATGGCGTCGAGTGACG	ThermoFisher	N/A
NO 2009 modification 2 AGA TAT CGC ATG CTT TTT AAA AGA CAT CAG AGA AAA AGA AAG ATA A	ThermoFisher	N/A
SY 2012 modification 1 AGA TAT CGG ATC CTA TAA ATA TGA AGA TGG CGT CGA GTG ACG C	ThermoFisher	N/A
SY 2012 modification 2 AGA TAT CCT GCA GTT TTT AAA AGA CAC TAA AGA AAA AGA AAG ATA A	ThermoFisher	N/A

**Recombinant DNA**		

pVR21 with norovirus capsid inserted	R. Baric, UNC	N/A
pRN16 with norovirus capsid inserted	D. Allen, LSHTM	N/A
Bacmid	D. Allen, LSHTM	N/A
pCR2.1-Topo	Thermo-Fisher	K455040

**Software and algorithms**		

Graphpad Prism v9.2	https://www.graphpad.com/scientific-software/prism/	RRID: SCR_002798
R version 4.1.3	https://www.R-project.org	RRID: SCR_002931
racmacs package	https://github.com/acorg/Racmacs	N/A
JAGS	Plummer, 2003^93^	RRID: SCR_017573
Biorender-graphical abstract	Biorender	https://biorender.com/
Antigenic cartography script	This paper.	https://zenodo.org/badge/latestdoi/518927355
ggplot2	Wickham, 2016^94^	RRID: SCR_014601

### Resource availability

#### Lead contact

Further information and requests for resources and reagents should be directed to and will be fulfilled by the lead contact, Ralph Baric (rbaric@email.unc.edu).

#### Materials availability

Materials generated in this study are available by MTA. All human serum samples remain property of the Public Health England Seroepidemiology Unit.

### Experimental model and subject details

#### Humans subjects

##### Serum samples

The Public Health England Vaccine Evaluation Unit (Manchester University NHS Foundation Trust) specializes in serological determination of immune responses and houses the Public Health England Seroepidemiology Unit (PHE SEU), part of the Serum Archive Section. The PHE SEU is an opportunistic collection of residual serum samples from routine microbiological testing, submitted voluntarily each year from hospital laboratories throughout England. For this study, approximately 700 sera from this archive were selected at random, sampled from the period 2008–2012 from children ages 1–6 years. The use of coded serum samples was approved by the National Health Service Research Ethics Committee (ref. 17/EE/0269), London School of Hygiene and Tropical Medicine (reference LEO12196), and the University of North at Carolina Chapel Hill (18–0214). All sera were received coded with no link back to donor identification. No demographic data other than age at serum collection were provided for this study. Sera were heat-inactivated for 30 min at 56°C and stored at −80°C until use. Subject numbers (n) for each analysis are reported in the corresponding legends.

#### Cell lines

BHK-21 cells (ATCC CCL-10) were derived from unsexed hamsters and grown at 37°C in 5% CO_2_ in Minimum Essential Medium with Earles salts (MEME) and L-glutamine (Gibco) supplemented with 7% fetal Clone II (Hyclone), non-essential amino acids (Gibco), sodium pyruvate (Gibco) and Antibiotic-Antimycotic (Gibco). Sf9 cells were maintained at 27°C in suspension cultures in Insect-XPRESS (Lonza) medium supplemented with 2% fetal bovine serum (ThermoFisher) and 1% antibiotic-antimycotic (Thermo-Fisher). The cell lines were not independently authenticated for these studies.

#### Microbe strains

*E coli* Top 10 containing pVR21 plasmids with norovirus capsid genes were propagated at 30°C in Luria-Bertani broth supplemented with 0.1 mg/ml carbenicillin.

### Method details

#### Production of virus like particles

For this study, NO 2009 (MZ376650) and SY 2012 (MZ376651) VLP were produced from baculovirus vectors as described.^95^^,^^96^ Two human norovirus positive stool samples, either containing GII.4 NO 2009 or GII.4 SY 2012 strains, were prepared as a 10–20% (v/v) fecal suspension in Medium 199 (Fisher Scientific), from which total nucleic acid was purified. Copy DNA (cDNA) comprising partial ORF1, complete ORF2+ORF3 and 3′ untranslated genome regions were generated using the SuperScript™ III Reverse Transcriptase System (Fisher Scientific) with primer 5′- NTTTTTTTTTTTTTTTTTTTTCGACCTAGGTGATACATGAT-3′. The cDNA was amplified with the Roche Expand High Fidelity System (Sigma Aldrich) to amplify from the ORF1/2 junction to the 3′ untranslated region (UTR) using forward primer 5′-CTGAGCACGTGGGAGGGCG-3′ and reverse primer 5′-CGACCTAGGTGATACATGAT-3′. Each amplicon of the expected size was purified from agarose gels with the Minelute Gel Extraction kit (Qiagen), following the manufacturer’s instructions. The purified amplicons were cloned into the intermediate vector pCR2.1-TOPO (Fisher Scientific) and clones were sequence verified. Each plasmid was modified by PCR to include *Sac*I/*Sph*I and *BamH*I/*Pst*I sites at the 5′ AND-3′ ends of the amplicon on the NO 2009 and SY 2012 sequences, respectively (NO 2009 primers: forward, 5′- AGATATCGAGCTCTATAAATATGAAGATGGCGTCGAGTGACG-3′, reverse 5′- AGATATCGCATGCTTTTTAAAAGACATCAGAGAAAAAGAAAGATAA-3′; SY2012 primers: forward 5′- AGATATCGGATCCTATAAATATGAAGATGGCGTCGAGTGACGC-3′, reverse 5′-AGATATCCTGCAGTTTTTAAAAGACACTAAAGAAAAAGAAAGATAA-3′). Sticky end ligation was then performed into pRN16 (a vector containing overlapping regions with *Autographa californica nuclear polyhedrosis virus*) and clones were sequence verified. The modified pRN16 and a bacmid were transfected into Sf9 cells with Cellfectin (Fisher Scientific), following the manufacturer’s instructions. Recombinant baculoviruses were plaque purified and used in a 3 MOI infection of 400 mL liquid culture Sf9 cells at 1×10^6^ cells per mL. After 72 h, cells were pelleted and lysed by suspending pellets in PBS buffer containing 1% IGEPAL (Sigma Aldrich) and 1X Protease Inhibitor Cocktail (Fisher Scientific), followed by Dounce homogenization. The cell lysates were layered onto a 15% w/v sucrose cushion which was layered onto a 60% w/v sucrose cushion (prepared in PBS). VLP were purified into the cushion by ultracentrifugation at 135,000×g for 3 h and stored at −20°C in sucrose/PBS solution. VLPs were verified by electron microscopy visualization for ∼40 nm particles.

FH 2002 (JQ478408)^23^ and DH 2006 (JQ478409)^58^ VLP were produced from Venezuelan equine encephalitis virus (VEE) vectors as described.^97^ Briefly, capsid encoding genes were prepared for insertion into the VEE cDNA plasmid pVR21 by *Apa*1/*Asc*1 (both NEB) digestion and ligation by the addition of GGGCCCCTATAACTCTCTACGGCTAACCTGAATGGACTACGACATAGTCTAGTCCGCCAAG at the 5-prime end and an *Asc*1 site at the 3-prime end. pVR21 containing norovirus capsid gene pDNA was purified with QIAprep Miniprep kit (Qiagen), linearized with *Not*I (NEB), gel purified with QIAquick Gel Extraction kit (Qiagen) and capped mRNA produced using the mMESSAGE mMachine T7 transcription kit (Invtirogen). mRNA was electroplated into BHK cells (ATCC CCL-10). At ∼27 h washed cells were lysed with 1% Triton X-100 in PBS supplemented with Roche cOmplete EDTA-free protease inhibitor cocktail (Sigma Aldrich). VLP were purified by ultracentrifugation at 120,000 x g for 75 min and stored at −80°C in 40% sucrose/PBS. Protein concentration was determined by BCA assay (Pierce) and particle integrity was confirmed by ligand and antibody binding and visualization by electron microscopy of ∼40nm particles.

#### Surrogate neutralization assay

Antibody blockade of VLP-ligand binding assays were performed as described.^39^ VLP (0.25 μg/mL) were pretreated with 2-fold serial dilutions of sera for 1 h and then added to pig gastric mucin type III (10 μg/mL in PBS, Sigma Aldrich) coated plates for 1 h. Bound VLP were detected by anti-VLP rabbit hyperimmune sera (Cocalico) followed by anti-rabbit IgG-HRP (Cytiva) and color developed with 1-Step Ultra TMB ELISA HRP substrate solution (Thermo-Fisher). Percent control binding was defined as the ratio of VLP binding in the presence of antibody pretreatment compared to the binding in the absence of pretreatment multiplied by 100. Each serum sample was tested in at least one 10-point dilution series on a plate that included a positive control. If a sample had an R^2^ < 0.85 for the model fit or if the control serum was outside the established range, the sample was repeated.

### Quantitaion and statistical analysis

All curve fit analyses were completed in GraphPad Prism V9.2 using dose-response sigmoidal curve fit of normalized data.^39^ Reported metrics include the Inhibitory Dose 50% titer (ID50), Hill slope, and 95% confidence intervals (95% CI). ID50 below the lowest dilution tested (10) were assigned a titer equal to ½ the limit of detection (in this case 5) for statistical comparisons. Log_10_ ID50 titers were compared by ANOVA with Dunnett multiple comparison test within year or VLP and by Mann-Whitney between a year or VLP. The Hill slope of the neutralization curve was reported as the relative avidity and differences were compared by ANOVA. Antigenic distances between variants within years or between years and within a variant were compared by ANOVA with Dunnett multiple comparison test. p < 0.05 was considered significant.

#### Antigenic cartography

The antigenic cartography analyses were carried out in R version 4.1.3 (https://www.R-project.org/) with the antigenic and sera coordinates calculated through the racmacs package (https://github.com/acorg/Racmacs) and the graphic displays done with ggplot2.^94^ The samples were grouped by age and year, and each round of analysis focused on highlighting the clustering of samples based on either age or year. The coordinates of the sera and antigens were calculated with a run of 1000 optimizations to minimize the difference between the n-dimensional Euclidean distances between points, and the two-dimensional distances on the final map. This process was repeated for both the neutralization data and the relative avidity data for any sample with at least two data points. For neutralization data this includes any sample with at least one neutralizing titer above the limit of detection (ID50 ≥ 10). A titer of 5 (less than the limit of detection) may be included for additional points) (Table S3), and for the avidity data this includes any sample with at least two neutralizing titers above the limit of detection (titer below the limit of detection does not have an associated slope) (Table S4). ID50 and relative avidity (slope) data and calculated antigenic distances are available at http://zendo.org/badge/latestdoi/518927355.

#### Catalytic model of response rates

To explore the response rate of children by age, year of sample collection and GII.4 variant we use a catalytic model where the probability of responding to a specific norovirus variant is a function of the child’s age, and we explore whether there is evidence for an upper limit of responding. For a child of age a, the probability of responding (p) is defined as follows;

$$p\left(a\right)=u-e^{-\lambda a}$$

With parameters being; λ - the rate of response (per year), u – the upper limit of the proportion that respond. The aggregated data consisted of x individuals of total n that responded to each variant by a, and so the data were assumed to be binomially distributed with *p* being the probability of response. An implicit assumption of the model is that there is no waning of response with age. The parameters of the model were estimated using a Gibbs sampler (5,000 iterations with a burn-in of 1,000 and 3 chains) and implemented in JAGS.^93^ The parameter u was examined using a hypothesis approach, if the 95% credible intervals exclude 0.999 then it suggests the presence of an upper limit, and also the deviance information criteria were compared between and model with u = 1.00 and a model where u was estimated.

## Data Availability

• Sera ID50 and relative avidity (slope) data and calculated antigenic distances have been deposited at http://zendo.org/badge/latestdoi/518927355 and are publicly available as of the date of publication. DOIs are listed in the key resources table. • All original code has been deposited at http://zendo.org/badge/latestdoi/518927355 and is publicly available as of the date of publication. DOIs are listed in the key resources table. • Any additional information required to reanalyze the data reported in this paper is available from the lead contact upon request.

## Supplemental information

Document S1. Figures S1–S4 and Tables S1–S4

Document S2. Article plus supplemental information
