# Supplementary material for: Emergent variant modeling of the serological repertoire to norovirus in young children
Source: Cell Rep Med. 2023 Feb 27;4(3):100954. doi: 10.1016/j.xcrm.2023.100954 (PMC10040388; doi:10.1016/j.xcrm.2023.100954)
Supplement: Document S1. Figures S1–S4 and Tables S1–S4 [file mmc1.pdf]

Cell Reports Medicine, Volume *4*

Supplemental information

Emergent variant modeling of the serological repertoire to norovirus in young children

Lisa C. Lindesmith, Paul D. Brewer-Jensen, Helen Conrad, Kathleen M. O'Reilly, Michael L. Mallory, Daniel Kelly, Rachel Williams, W. John Edmunds, David J. Allen, Judith Breuer, and Ralph S. Baric

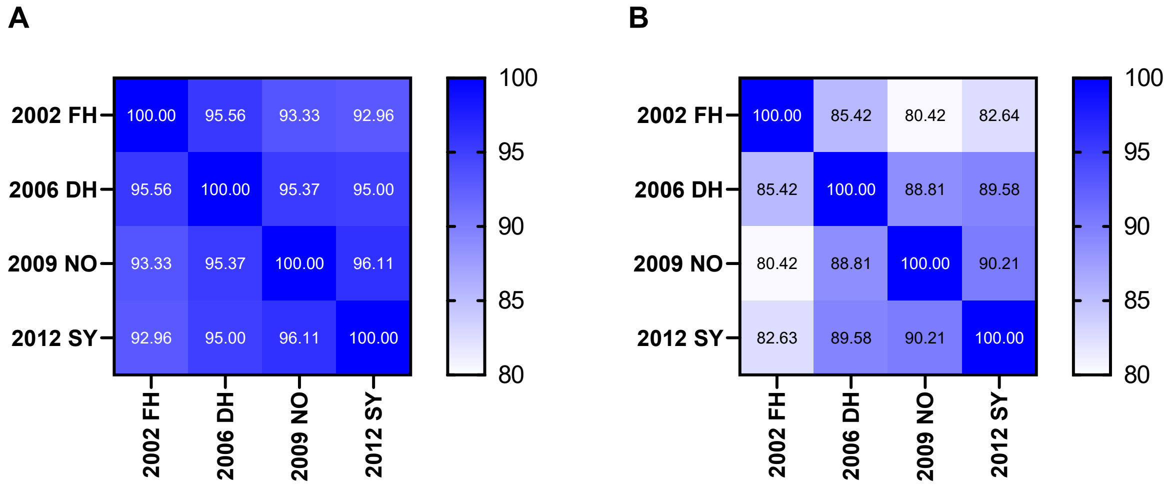

Figure S1. Percent amino acid identity between the study variants. Related to Figure 1. The amino acid sequences of VP1 **(Panel A)** or the VP1 P2 subdomain **(Panel B)** were aligned in Geneious Prime v2022.2.2 using Geneious Alignment default parameters and the percent identity tabulated. Hypervariable nAb epitopes map to the VP1 P2 subdomain.

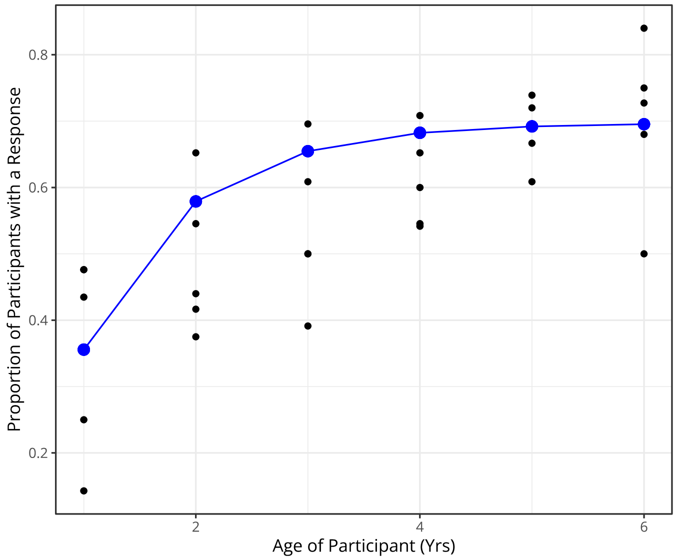

Figure S2. Proportion of children with a response to the GII.4 FH 2002 variant by age (black dots) and the modelled fit (blue line) to the data using a catalytic model. Each black dot represents the proportion with a response for each year (2008-2012) where blood samples were taken. Related to Figure 2.

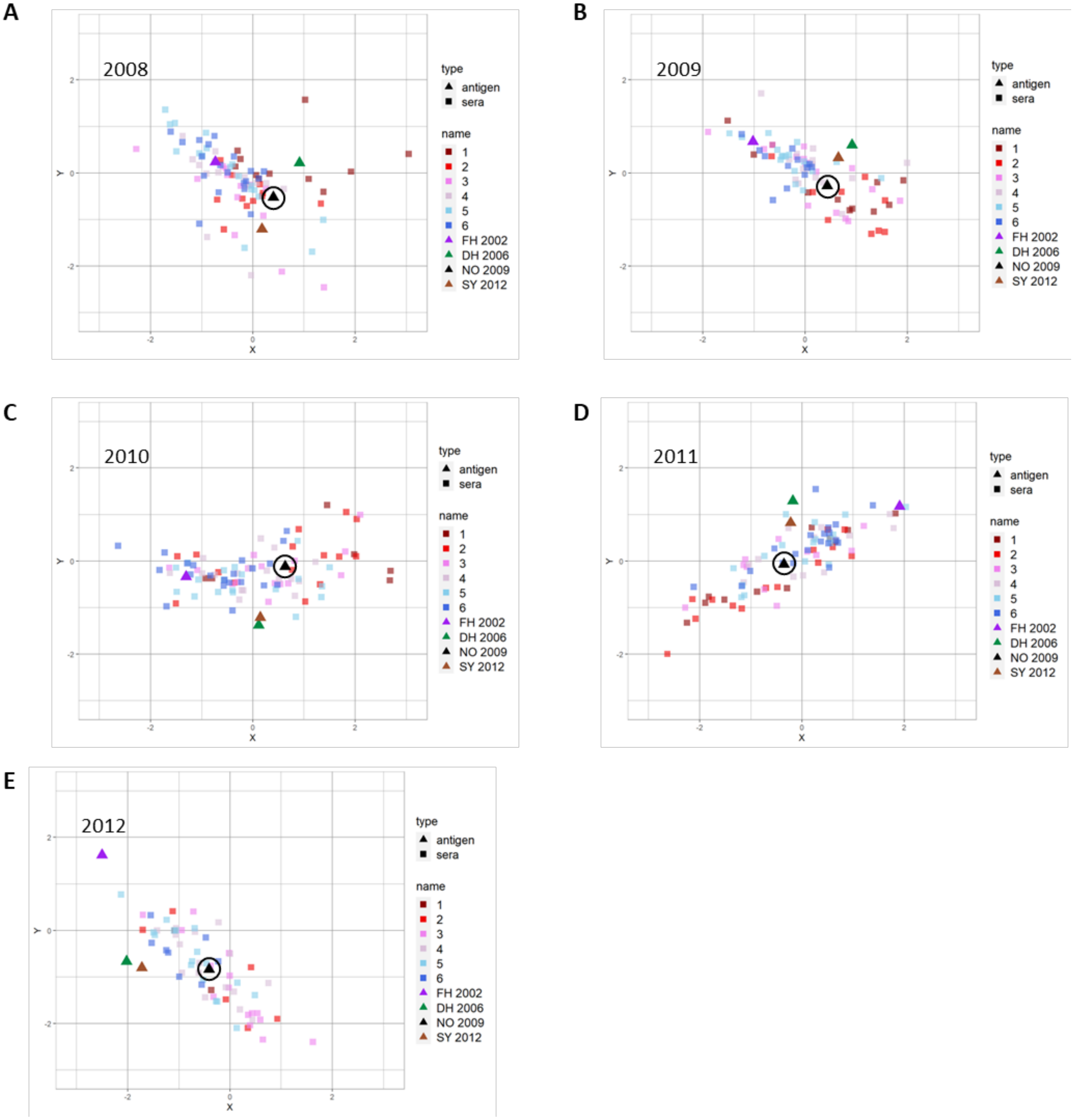

Figure S3. Antigenic distances between GII.4 variants defined by nAb titer. Related to Figure 5. **Panels A-E:** The antigenic relationship between GII.4 variants (triangles) relative to the nAb response in each child's serum sample with measured titer to at least two GII.4 variants (squares, n=413, Table S3) color-coded by year of age (color of square: 1-dark red, 2-red, 3-pink, 4-light pink, 5-light blue, 6-dark blue) were mapped via antigenic cartography for years 2008-2012 (**Panels A-E**). As a visual marker, NO 2009 is circled. One grid box (antigenic distance unit, AD) corresponds to a 2-fold change in titer.

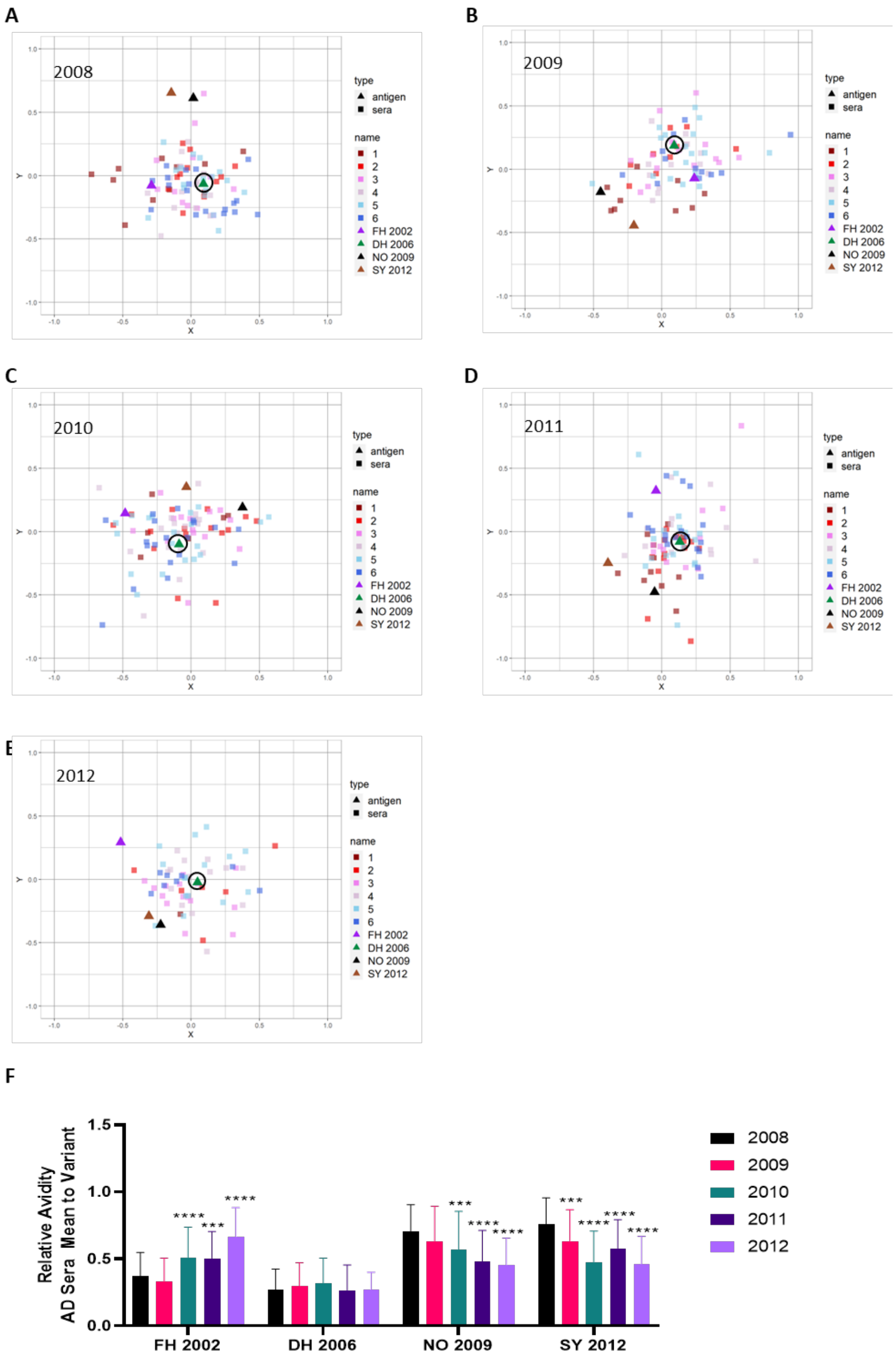

Figure S4. Antigenic distances between GII.4 variants defined by nAb relative avidity. Related to Figure 6. **Panels A-E:** The antigenic relationship between GII.4 variants (triangles) defined by nAb relative avidity in serum from each child with measurable titer to at least two GII.4 variants (n=394, **Table S4**) (squares) color-coded by year of age (color of square: 1-dark red, 2-red, 3-pink, 4-light pink, 5-light blue, 6-dark blue) were mapped via antigenic cartography for 2008 - 2012. One grid box (antigenic distance unit, AD) corresponds to a 2-fold change in avidity. As a visual marker, DH 2006 is circled. One grid box (antigenic distance unit, AD) corresponds to a 2-fold change in avidity. **Panel F:** Antigenic distances (AD) between variants and the sera mean in Panels A – E. Bar and error, mean and standard deviation. ***, $P \leq 0.001$. ****, $P \leq 0.0001$ compared to 2008 (One-way ANOVA, Dunnett Multiple comparison test).

		Parameter estimates						
**nAb**	**Model**	**Lambda**			**U**			**DIC**
		Median	Lower	Upper	Median	Lower	Upper	Estimate
FH 2002	Null	0.255	0.228	0.282				150.4
FH 2002	‘u’ estimated	1.17	0.763	1.64	0.646	0.588	0.705	137.6
DH 2006	Null	0.254	0.228	0.281				154.1
DH 2006	‘u’ estimated	1.18	0.797	1.65	0.643	0.587	0.7	137.2
NO 2009	Null	0.264	0.237	0.264				153.7
NO 2009	‘u’ estimated	1.25	0.834	1.76	0.651	0.597	0.706	133.6
SY 2012	Null	0.257	0.231	0.285				146.4
SY 2012	‘u’ estimated	1.09	0.752	1.51	0.656	0.599	0.715	131.6
**Any norovirus**	Null	0.289	0.26	0.319				151.9
**Any norovirus**	‘u’ estimated	1.08	0.721	1.51	0.697	0.64	0.759	135.5

Table S1. Summary of parameter estimates for the catalytic model fitted to the response data. Related to Figure 2.

Age	2008	2009	2010	2011	2012	Total
1.0-1.9	11	11	6	12	2	42
2.0-2.9	11	9	15	15	6	56
3.0-3.9	13	17	15	14	15	74
4.0-4.9	16	15	18	14	16	79
5.0-5.9	18	19	19	17	18	91
6.0-6.9	19	12	21	21	8	81
**Total**	88	83	94	93	65	423

Table S2. Number of responders to any GII.4 variant included in Figures 3 and 4 (minimum of one GII.4 variant titer above the limit of detection). Related to Figures 3 and 4.

Age	2008	2009	2010	2011	2012	Total
1.0-1.9	11	11	6	11	2	41
2.0-2.9	11	9	15	14	6	55
3.0-3.9	13	16	15	11	15	70
4.0-4.9	16	15	18	13	16	78
5.0-5.9	18	18	19	17	17	89
6.0-6.9	19	12	21	20	8	80
Total	88	81	94	86	64	413

Table S3. Number of responders included in cartography analysis of GII.4 variant nAb titer in Figures 5 and S3 (minimum of two reported titers/subject are required to map a serum sample). Related to Figure 5.

Age	2008	2009	2010	2011	2012	Total
1.0-1.9	11	9	6	11	1	38
2.0-2.9	11	9	15	13	6	54
3.0-3.9	12	15	14	11	15	67
4.0-4.9	15	14	18	12	15	74
5.0-5.9	17	17	19	17	15	85
6.0-6.9	18	12	20	18	8	76
**Total**	84	876	92	82	60	394

Table S4. Number of responders included in cartography analysis of GII.4 variant nAb avidity in Figures 6 and S4 (minimum of two reported titers above the limit of detection/subject are required to map a serum sample by avidity). Related to Figure 6.
